# Functional Movement Screening and Paddle-Sport Performance

**DOI:** 10.3390/sports5020037

**Published:** 2017-06-13

**Authors:** Andrew Hatchett, Charles Allen, Jake St. Hilaire, Alex LaRochelle

**Affiliations:** 1 Exercise and Sports Sciences, University of South Carolina Aiken, Aiken, 29801 SC, USA; 2 Exercise Science, Florida Southern College, Lakeland, 33801 FL, USA; callen@flsouthern.edu; 3 Health Science, Franklin Pierce University, Rindge, 03461 NH, USA; st.hilairej12@gmail.com (J.S.H.); Larochellea12@live.franklinpierce.edu (A.L.)

**Keywords:** functional movement screening, endurance, paddle, canoe, kayak, performance

## Abstract

The purpose of the study reported here was to determine the relationship between an endurance paddle-sport athlete’s total functional movement screening (FMS) score and individual race performance. Fifty elite level endurance canoeists and kayakers completed the seven-stage FMS protocol prior to the 2016 United States Canoe and Kayak Association National Championship race. Time taken to finish the race was then associated to overall FMS score and respective sub-scores. Total FMS score and various sub-scores were significantly related to race performance. Female and male athletes differed in which sub-scores were shown to be significantly correlated to finishing time. Outcomes from this study indicate that limitations in functional movement are related to endurance paddle-sport race performance.

## 1. Introduction

The International Canoe Federation defines a canoe and/or kayak marathon as an event in which the competitor races over a designated long distance course, on water, and subject to prescribed standards. The competitor must take the water as it is found and be prepared, if it is necessary, to carry his or her canoe around an impassable obstacle, or between two waterways [1]. Endurance paddle-sport contests can range from 13 to 170 miles over several days and include sections of portage [2]. These rules and standards are generally held consistent for other paddle sports such as marathon kayak racing as well. Marathon paddling is a low-impact, high-aerobic sport [3]. The sport also has significant technical components, which include paddling techniques, water knowledge, and navigation skills [4]. Over time, athletes develop the ability to read different water conditions so as to both avoid obstacles and seek out faster-moving water [3].

Just as optimal running form requires a certain level of stability and mobility in order to perform specific movement patterns to achieve efficiency [4], so does an optimal paddling technique [5]. Similarities between running and paddling from a mechanical perspective are many. Both running and paddling utilize the entire human machine to successfully perform an effective and efficient movement pattern. Running form has been assessed numerous times with a multitude of methods and criteria to determine areas of influence on performance [6]. Conversely, the optimal stroke profile for paddle-sport athletes has considerably less evaluation [7].

Previous research has determined that the FMS assessment protocol is a reliable baseline functional screen for long-distance athletes [5]. The FMS is a comprehensive screen to assess the quality of fundamental movement patterns for presumably identifying an individual’s physical limitations or asymmetries [8]. Research has indicated that FMS scores are associated injury rates of various athlete populations [9,10,11,12,13,14,15,16,17,18,19,20,21,22]. Conversely, other research has determined the FMS does not sufficiently predict the risk of injury in specific performance populations and questions the tool’s accuracy [23,24,25]. Intuitively, a movement limitation or asymmetry in an athlete can translate to a compromised event performance due to an inability to perform efficiently. This may also hold true for athletes that participate in events that require negotiation of diverse and unique obstacles, such as paddle-sport athletes. The researchers hypothesized that if an endurance paddle-sport athlete were to have compromised functional movement, per their FMS score, their race performance would be adversely affected.

Therefore, the purpose of the study reported here was to determine the relationship between an endurance paddle-sport athlete’s total FMS score and sub-score assessments, and race performance.

## 2. Materials and Methods

### 2.1. Study Design

USCA Marathon National Championship athletes are required to either register or check-in prior to the respective event they intend to compete throughout the four-day event. A team of United States Canoe Association officials reviewed and approved the data collection procedures proposed by the researcher in a manner consistent with other endurance events. All data collection procedures were consistent with criteria detailed by the Center for Disease Control and Prevention Human Participant Protection standards.

### 2.2. Participants

Fifty endurance paddle-sport athletes in 2016 United States Canoe Association (USCA) Marathon National Championships participated in this research. The 2016 USCA Marathon National Championships took place in Northfield, Massachusetts, USA, between August 11 and August 14 on the Connecticut River. The course for this year’s race was a 13-mile looped course that afforded athletes the opportunity to compete in a number of different events over the four days of competition. These events included individual, pair, and mixed-pair, events. This event attracts some of the finest endurance canoeists and kayakers in the United States of America and Canada, allowing them to compete head to head [1].

### 2.3. Data Collection

All data collection took place during athlete check-in/registration during the championship event. After providing written informed consent, the participant’s age, weight, height, and body composition were measured [26]. Based upon the individual’s height and weight, body mass index was then calculated. The study participant then completed the FMS protocol with a researcher well versed in, and experienced with, the protocol. Individual researchers were assigned to specific segments of the data collection process in an effort to insure consistency and inter-rater reliability.

### 2.4. Measures

The FMS as described by Cook et al. [22] was used in the study. The FMS consists of seven fundamental movement patterns to test mobility and stability [8]. The seven tasks are the deep squat, hurdle step test, in-line lunge, shoulder mobility, active straight leg raise (SLR), push-up, and rotary stability. Further description can be found in several resources [27,28]. Performances were scored using standardized FMS criteria. The scoring criterion for each of the seven tests is a 4-point scale (0–3). A score of 3 was awarded for perfect form (normal functional movement pattern), a 2 for completing the test with compensations, a 1 for not completing the test accurately, and a 0 if the subject noted any pain with the testing components. For the tests that were assessed bilaterally, the lowest score was used. The maximal score that can be achieved is 21. Each movement was practiced and then performed again for scoring, in the same standardized order, and recorded on a score sheet. If the examiner was not sure about the scoring, then the movement was repeated. No warm-up was included. Additionally, researchers recorded bilateral scores for the hurdle step test, in-line lunge, shoulder mobility, active straight leg raise, and rotary stability. This additional data was intended to determine if a hemispheric discrepancy existed within athletes and its influence on race performance.

### 2.5. Statistical Analysis

Race results offered by race officials were used in the analysis of the relationship between FMS data and athlete race performance. Pearson correlations were used to determine the relationship between FMS assessment values with a finish time in the individual distance contest. Fisher’s z transformation, 95% percentile confidence intervals (upper and lower limits), mean values, and standard deviations were also determined. Quartiles were determined allowing for additional correlational analysis to be conducted. All calculations were performed using SPSS (Version 24) with an a priori level of significance set at *p* ≤ 0.05 and *p* ≤ 0.01, respectively.

## 3. Results

Characteristics of the participants in this study as a pooled sample are presented in Table 1. A total of 252 athletes competed in categories designating individual canoe or kayak classes. Of the 252 athletes, a total of 50 athletes participated in this study (19%). Of the 50 athletes who participated in data collection, 15 were female (30%) and 35 were male (70%), which approximately reflects the overall event participation (72% male and 28% female). Mean finish times of the study participants were comparable to all race participants finish times. Table 1 and Table 2 display anthropomorphic and Finish Time characteristics of the study participants.

Table 3 offers the mean values of the seven scoring categories of the FMS.

Significant correlational data including r value, z transformation, 95% confidence interval lower and upper limits for all athletes between FMS score (and sub-scores) and finish time are reported in Table 4.

The mean composite score for all participants for FMS was 13.20 (±3.14) with an interquartile range of 4 (Q1 = 11, Q3 = 15). Significant correlational data including r value, z transformation, 95% confidence interval lower and upper limits between FMS score (and sub-scores) and finish time, by participants scoring 13 or greater on the total FMS score are reported in Table 5.

Athletes earning a composite FMS score of less than 13 did not report significant correlations with any FMS score or sub-score.

## 4. Discussion

The FMS is a diagnostic tool that can be used to assist in the determination of limitations that are likely to lead to injury of an athlete. The results of this study indicate there is a significant relationship between FMS score and finish time for endurance paddle-sport athletes. Those athletes earning a total FMS score of 13 or greater displayed significant relationships with finish time, several FMS sub-scores, along with total FMS. Additionally, endurance paddle-sport athletes that report a FMS score of less than 13 did not display a significant relationship with finish time, any FMS sub-score, or total FMS score. These results suggest that a variety of functional movement capacities can have meaningful influence on endurance paddle-sport race performance.

Areas of assessment identified in this study with significant influence on endurance paddle-sport performance are the in-line lunge, straight leg raise, trunk stability, and rotary stability. Each of these respective areas is related to midline or core stabilization. The in-line lunge is a test that places the lower extremity in a scissored position, challenging the body’s trunk and extremities to resist rotation and maintain proper alignment. This test assesses torso, shoulder, hip, and ankle mobility and stability, quadriceps flexibility, and knee stability. The active straight-leg rise test assesses active hamstring and gastrocnemius-soleus flexibility, while maintaining a stable pelvis and active extension of the opposite leg. The ability to perform the active straight-leg raise test requires functional hamstring flexibility, which is the flexibility that is available during training and competition. This is different from passive flexibility, which is more commonly assessed. The subject is also required to demonstrate adequate hip mobility of the opposite leg as well as lower abdominal stability. The trunk stability push-up tests the ability to stabilize the spine in an anterior and posterior plane during a closed-chain upper body movement. The ability to perform the trunk stability push-up requires symmetric trunk stability in the sagittal plane during a symmetric upper extremity movement. Many functional activities require the trunk stabilizers to transfer force symmetrically from the upper extremities to the lower extremities and vice versa. If the trunk does not have adequate stability during these activities, kinetic energy will be dispersed, leading to poor functional performance as well as increased potential for micro-traumatic injury. The rotary stability test assesses multi-plane trunk stability during a combined upper and lower extremity motion. The ability to perform the rotary stability test requires asymmetric trunk stability in both sagittal and transverse planes during asymmetric upper and lower extremity movement. Many functional activities require the trunk stabilizers to transfer force asymmetrically from the lower extremities to the upper extremities and vice versa. If the trunk does not have adequate stability during these activities, kinetic energy will be dispersed, leading to poor performance as well as increased potential for injury [22].

The midline or core is where the body’s center of gravity is located and where all movement originates. A strong and efficient core is necessary for maintaining proper muscle balance throughout the entire human movement system (kinetic chain). Optimal length–tension relationships, recruitment patterns, and joint motions in muscle of the lumbo–pelvic–hip complex (LPHC) establish neuromuscular efficiency throughout the entire human movement system, allowing for efficient acceleration, deceleration, and stabilization during dynamic movements, as well as the prevention of possible injuries. Muscles within the LPHC attach from pelvis to the spine. These transfer loads between upper extremity and lower extremity, provide stability between pelvis and spine, and provide stabilization and eccentric control of the core during functional movements [29].

The areas identified with significant relationships to race performance in this study and the influence midline stabilization can have within the kinetic chain of a paddle stroke are consistent with the results reported by Abraham and Stepkovich [30], as well as the results of others examining the injury history of paddle-sport athletes [31,32,33]. Abraham and Stepkovich conducted a study that determined the areas of injury most commonly reported by paddle-sport athletes are the shoulder and back [30]. Abraham and Stepkovich [30] articulate well that previous research conducted by Hagemann et al. [33] investigated marathon kayakers’ shoulders and found that 22 of 52 marathon kayakers had symptoms of shoulder pain and instability. Abraham and Stepkovich go on to report that Kameyama et al. investigated the most common pathologies in paddlers [30]. Kameyama et al. reported that 25% of respondents complained of concurrent shoulder and lower back pain [31]. After diagnostic investigations on competitive canoeists, the researchers noted that 52.3% of competitors complained of lower back pain, spondylosis deformans, and disc herniation. Furthermore, Humphries et al. [34] assessed physiologic characteristics of outrigger canoeists and advised that these athletes should be concerned with the muscular strength imbalances associated with paddling technique. Based on the results of this study, the same advice may hold true for endurance paddle-sport athletes. Although the present study did not attempt to determine the injury history of the participants and was focused on the relationship between functional movement and race performance, it is important to recognize the likelihood that an athlete develops an injury in the areas in which limitations have been identified.

The results determined from this study indicate a significant relationship between a higher total FMS score and finish time, but more importantly a significant relationship between several FMS sub-scores that indicate functional capability in areas directly related to the LPHC and the kinetic chain of a paddle stroke. Additionally, as the paddle stroke is analyzed, the generation of power is centered on the hip drive, translating the chain of force through a functional and stable midline to the shoulder in an effort to complete the stroke. Any limitation in the links of this kinetic chain may result in a decrease in power, a decrease in efficiency, and/or lead to injury of the back or shoulder as a result of requiring these joints to generate inappropriate force. These reports are also consistent with the results found in the current study showing a significant relationship between specific elements of the FMS screening protocol associated with the back and related to shoulder and race performance. It was determined that a significant relationship between higher performing athletes (in terms of lunge, leg raise, rotary stability, and trunk stability) and race performance was present. These results corroborate reports by other researchers [30,31,32,33,34] indicating disorders of the back and shoulders as having the highest rate of report injury.

Functional movement screening can be a useful evaluative tool for identifying asymmetries in movement patterns of endurance paddle-sport athletes. Based on the results of this research, a significant relationship exists between total score and specific sub-scores during FMS and endurance paddle-sport race performance. Future research should investigate the relationship between paddle-sport athlete FMS scores and reported injury history. Based upon this information, stroke pattern and conditioning protocols could be developed in an attempt to rectify asymmetries in an effort to enhance efficiency of movement, resulting in better performance.

## Figures and Tables

**Table 1:** Characteristics of race participants (*n* = 50).

Variable	Minimum	Maximum	Mean	SD
Age (yrs)	21	94	53.96	17.37
Height (cm)	156.21	193.04	173.58	8.92
Weight (kg)	55.34	115.67	75.11	12.71
BMI (kg/m^2^)	20.41	32.21	24.85	2.92
Body Fat %	6.41	35.61	22.21	6.31
Finish Time (min)	119.15	169.57	135.75	13.33

**Table 2:** Characteristics of race participants by sex.

Participant Sex	Variable	Minimum	Maximum	Mean	SD
Female (*n* = 15)	Age (yrs)	23.00	76.00	50.86	17.26
	Height (cm)	156.21	177.80	165.59	6.60
	Weight (kg)	55.79	85.00	63.81	7.48
	BMI (kg/m^2^)	20.40	32.20	23.72	2.98
	Body Fat %	17.01	35.61	25.86	6.47
	Finish Time (min)	119.65	154.45	133.99	11.52
Male (*n* = 35)	Age (yrs)	21.00	94.00	55.14	18.02
	Height (cm)	160.02	193.04	176.81	7.87
	Weight (kg)	55.34	115.67	79.78	11.88
	BMI (kg/m^2^)	20.40	32.00	25.32	2.89
	Body Fat %	6.41	32.10	20.82	5.83
	Finish Time (min)	119.15	169.57	135.98	14.27

**Table 3:** Mean values of functional movement screening (FMS) scores.

Variable	Mean	SD	Range
Deep Squat	1.690	0.713	0–3
Inline Lunge	1.160	0.138	0–3
Shoulder Mobility	1.675	0.987	0–3
Hurdle Step	1.694	0.098	0–2
Straight Leg Raise	2.408	0.609	0–2
Trunk Stability	2.082	0.073	0–3
Rotary Stability	1.898	0.073	0–3
Composite Score	13.200	3.142	0–21

**Table 4:** Significant correlational data between FMS and finish time.

Variable	Significance	R Value	Z Transformation	95% CI Lower	95% CI Upper
Inline Lunge Left Leg	0.045	0.346	0.361	0.089	0.583
Inline Lunge Right Leg	0.047	0.339	0.353	0.080	0.576
Straight Leg Raise Left	0.041	0.348	0.363	0.092	0.584
Straight Leg Raise Right	0.034	0.361	0.378	0.109	0.595
Straight Leg Raise Total	0.041	0.348	0.363	0.092	0.584
Rotary Stability Left	0.007	0.450	0.485	0.236	0.674
Rotary Stability Right	0.003	0.490	0.536	0.301	0.710
Rotary Stability Total	0.001	0.558	0.630	0.424	0.774
Total FMS	0.015	0.407	0.432	0.172	0.635

**Table 5:** Significant correlational data between FMS and finish time by total FMS score of 13 or greater.

Variable	Significance	R Value	Z Transformation	95% CI Lower	95% CI Upper
Inline Lunge Left	0.043	0.504	0.555	0.205	0.779
Inline Lunge Right	0.046	0.537	0.600	0.269	0.804
Inline Lunge Total	0.039	0.485	0.53	0.171	0.764
Straight Leg Raise Left	0.001	0.586	0.672	0.377	0.843
Straight Leg Raise Right	0.026	0.576	0.656	0.353	0.834
Straight Leg Raise Total	0.017	0.586	0.672	0.377	0.843
Trunk Stability	0.030	0.311	0.322	0.083	0.635
Rotary Stability Left	0.026	0.380	0.400	0.006	0.686
Rotary Stability Right	0.028	0.334	0.347	0.055	0.652
Rotary Stability Total	0.047	0.356	0.372	0.027	0.668
Total FMS	0.004	0.628	0.738	0.485	0.877
